# Electrocardiographic Repolarization Changes Following Orchiectomy: Insights Into QT Interval Prolongation and Clinical Implications

**DOI:** 10.1111/anec.70226

**Published:** 2026-08-09

**Authors:** Javeria Anees

**Affiliations:** ^1^ Bolan Medical College Quetta Pakistan

Dear Editor,

I read with considerable interest the article by Sakaguchi et al., recently published in the *Annals of Noninvasive Electrocardiology*, examining electrocardiographic repolarization changes following orchiectomy, with particular emphasis on QT and corrected QT (QTc) interval prolongation. This study addresses a clinically important and insufficiently characterized area of cardio‐oncology, and the authors are to be commended for their systematic approach to a topic with meaningful arrhythmic implications.

In this retrospective analysis of 44 male patients who underwent unilateral or bilateral orchiectomy, the authors report significant prolongation of both QT and QTc intervals following surgery, most pronounced in the bilateral orchiectomy subgroup (QTc: 418 ± 36 ms to 441 ± 37 ms, *p* < 0.001). QTc was derived exclusively using Bazett's formula, a methodological choice that, I respectfully contend, warrants critical scrutiny, as it carries material consequences for the validity of the study's principal outcome.

It is well established that Bazett's formula systematically overcorrects QTc at elevated heart rates and undercorrects at lower rates, introducing rate‐dependent artifact that can distort the apparent magnitude of repolarization change (Bazett 1920). The group‐level heart rate stability reported here (72 ± 16 vs. 72 ± 15 beats/min, *p* = 0.97) may mask substantial inter‐individual variability inherent in a cohort with a standard deviation of ±15–16 beats/min, a range across which Bazett's formula is well‐documented to produce rate‐dependent bias (Fridericia 2003).

At the individual level, patients who experienced even a modest heart rate increase at the time of post‐operative ECG acquisition, attributable to disease progression, comorbidities, or autonomic remodeling following androgen deprivation, Bazett's formula would yield a spuriously elevated QTc, potentially misattributing a rate‐mediated shortening of the RR interval as pathological QT prolongation. Without individual pre‐ and post‐operative heart rate data recorded at the time of ECG acquisition, it is not possible to quantify what proportion of the observed ΔQTc reflects genuine repolarization prolongation versus formula‐induced artifact. Concurrent application of Fridericia's cube‐root correction or the Framingham linear formula, both shown to outperform Bazett's method across a broad physiological range, would have provided essential corroborative evidence (Luo et al. 2004; Sagie et al. 1992; Rautaharju et al. 2009).

I therefore urge the authors to reanalyze their dataset using at least one validated alternative correction formula. Concordance of QTc prolongation across two independent methods would substantially reinforce the robustness of the findings and strengthen the clinical applicability of the recommendations advanced, particularly given the arrhythmic risk the authors appropriately highlight.

## Author Contributions

The author takes full responsibility for this article.

## Funding

The author has nothing to report.

## Disclosure

This letter has not been submitted elsewhere and is not under consideration by any other journal. The author confirms that the content is entirely original.

## Conflicts of Interest

The author declares no conflicts of interest.

## Data Availability

The author has nothing to report.
